# Ovarian Torsion in a Pet Rabbit (*Oryctolagus cuniculus*): A Case Report

**DOI:** 10.3390/ani14172475

**Published:** 2024-08-26

**Authors:** Emilio Noviello, Marco Russo, Paola Rubino, Daniela De Felice, Stefano Spada

**Affiliations:** 1 Centro di Recupero Animali Selvatici (CRAS), University of Naples, Federico II, 80145 Naples, Italy; emilionoviello@yahoo.it; 2 Department of Veterinary Medicine and Animal Production, University of Naples, Federico II, 80137 Naples, Italy; marco.russo@unina.it (M.R.); daniela.defelice2@unina.it (D.D.F.); 3 Clinica Veterinaria “NewPet”, Casoria, 80026 Naples, Italy; prubinovet@gmail.com

**Keywords:** ovary, ovarian torsion, rabbit, *Oryctolagus cuniculus*, whirlpool sign

## Abstract

**Simple Summary:**

Genital tract disorders are among the most prevalent conditions affecting female pet rabbits (*Oryctolagus cuniculus*). However, ovarian diseases are relatively rare, constituting only 3.3% of reproductive conditions in this species. These may include ovarian cysts, neoplasia, and necrosis. This study presents the first documented case of spontaneous ovarian torsion in rabbits. A female pet rabbit presented for routine clinical examination exhibited a large abdominal mass, the nature of which remained ambiguous upon diagnostic imaging. Computed tomography revealed a mass exhibiting specific signs indicative of torsion of the supporting ligaments of the right ovary. Confirmation of the diagnosis was achieved during laparotomy, where twisting of the right ovarian pedicle was observed. The rabbit was neutered, and the mass underwent histological examination, revealing predominantly necrotic areas with no evidence of neoplastic cells.

**Abstract:**

Ovarian torsion (OT) is a condition that can affect both humans and animals, although it is less common in the latter, with very few cases documented in the literature. To our knowledge, no previous reports have documented the occurrence of this condition in rabbits. In this study, we present the first documented case of spontaneous OT in a 2-year-old female intact rabbit. The patient was brought to the clinic for a routine check-up, during which a firm, large abdominal mass was palpated. Subsequent ultrasound examination of the abdomen revealed the presence of a large, hypoechoic, non-vascularized mass occupying the majority of the caudal abdomen. A computed tomography (CT) scan further confirmed the presence of a heterogeneous mass exhibiting the Whirlpool sign, which is characteristic of organ torsion. Upon laparotomy, an enlargement of the right ovary was observed, characterized by twisting of the ovarian pedicle, consistent with the mass detected via ultrasound and CT scan. Ovariohysterovaginectomy was performed, and the mass was subsequently analyzed. Microscopic analysis of the mass revealed predominantly necrotic tissue, with only a few ovarian epithelial cells present. The underlying cause of the OT described in this study remains unclear. However, it is plausible that a previous neoplastic condition or ovarian necrosis led to an increase in the size and weight of the mass, ultimately resulting in the twisting of the supporting structures.

## 1. Introduction

According to authors in both the United States and the United Kingdom, rabbits have become increasingly popular as pets [1,2], being ranked as the third most favored companion animal after cats and dogs in households across these countries [3,4]. The increase in the number of pet rabbits owned in the last decades necessitates a deepened understanding of their clinical care and management for both owners and vets.

A recent Canadian survey revealed a positive correlation between owners’ knowledge levels and the likelihood of having their rabbits neutered. This correlation is crucial for managing companion animal populations and ensuring rabbit health [5]. Ovariohysterectomy (OHE) is a common surgical procedure for female companion rabbits and offers benefits beyond preventing unwanted litters, including improved health, the prevention of serious reproductive diseases, and a reduction in undesirable behaviors [1,3]. Genital tract disorders are among the most common conditions of the female pet rabbit (*Oryctolagus cuniculus*), with uterine tumors being identified as the most frequent form of neoplasia [6,7]. A recent study evidenced that reproductive (65.08%) and integumentary (22.25%) systems are the most commonly affected by neoplastic changes [8]. In particular, uterine adenocarcinoma is the most frequent neoplasia occurring in female rabbits [8,9], showing a high potential to metastasize to the lungs, liver, brain, or bone [10]. Non-neoplastic uterine conditions such as endometritis and endometrial hyperplasia are also reported [11,12,13]. Uterine diseases in rabbits generally progress slowly, and affected rabbits may develop clinical signs of lethargy, anorexia, hematuria, serosanguineous vaginal discharge, mammary gland abnormalities, or urethral obstruction [10].

Ovarian diseases are less frequent, with ovarian cysts being the most frequent condition among them, and in 72.5% of cases, a concurrent non-inflammatory uterine disorder may occur [14]. Ovarian neoplasia, necrosis, hematoma, and oophoritis are also reported, even though their occurrence is extremely low. In particular, ovarian diseases may misshapen the affected ovary and determine compression and inflammation [14]. Although OT has been experimentally induced in rabbits for research purposes, spontaneous occurrences of this condition in the species have not been reported in the literature [15,16,17].

In the present study, we present a case of spontaneous OT in a female pet rabbit.

## 2. Clinical Case

A 2-year-old, 1.8 kg, intact female pet rabbit was presented to the clinic veterinary hospital for a routine clinical check-up and vaccination. The rabbit lived alone without other animals, maintained a regular and balanced diet, and received routine vaccinations and parasite prevention. During the general clinical examination, a large, solid oval intra-abdominal mass was detected at palpation, causing mild pain and discomfort to the patient. The owner did not report any remarkable clinical signs apart from mild lethargy, and all general parameters were within reference intervals. Popliteal and submandibular lymph nodes appeared normal at palpation, and the temperature was 39.3 °C.

Considering the patient’s history and the location of the mass, the following potential diagnoses were considered: a reproductive or non-reproductive neoplastic condition such as abdominal abscess, lipoma or liposarcoma, and trichobezoar.

Blood analysis, including a blood cell count (Idexx ProCyte Dx^®^ Haematology Analyser, IDEXX Laboratories, Inc., Westbrook, ME, USA) and a biochemistry profile (Abaxis Vetscan2, ©2020 Abaxis, Union City, CA, USA), was conducted according to the owner’s consent. Blood was collected via the lateral saphenous vein using a 24 Gauge, 1 mL syringe, following hair clipping and disinfection of the area with a 4% Chlorhexidine solution. Results from the blood analysis were unremarkable, as detailed in Table 1.

The patient underwent an abdominal ultrasound (Versana Active^TM^, GE Healthcare, Chicago, IL, USA), performed on right lateral recumbency and using a multifrequency microconvex transducer. Prior to the procedure, abdominal hair was clipped, and acoustic gel was applied to enhance the quality of the ultrasonographic image. No sedation was needed during the procedure as the rabbit was cooperative. During the ultrasound examination, a hypoechoic ovoid structure measuring 5.5 cm × 6.7 cm with homogeneous parenchyma was identified in the region of the right ovary. No signs of peritoneal reactivity or effusion were observed in the adjacent area of the mass. A Color Doppler evaluation was performed to assess vascularization of the mass, but no signs of vascular blood flow were detected either peri- or intranodularly, despite using a low PRF (pulse repetition frequency).

Both uterine horns appeared regular in size and outline, with preserved integrity of the layers and no endoluminal content. The left ovary exhibited regular morphology, echotexture, and normal vascularization.

**Figure 1:**
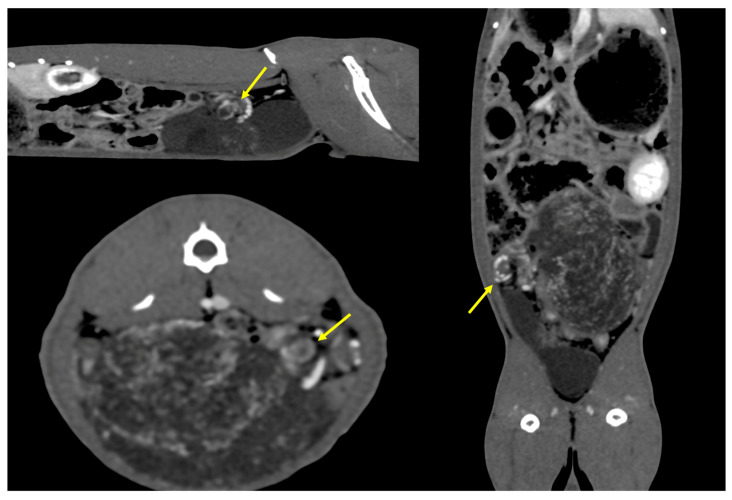
CT scan after contrast agent administration revealed a whirlpool sign detected in cases of organ torsion, consisting of a centrifugal vascular pattern (yellow arrows) laterally to the mass.

Based on the ultrasonographic findings, a CT examination was deemed necessary to further elucidate the nature and origin of the mass. All scans were performed using a 128-slice CT scanner with the administration of an intravenous ionic non-iodinated contrast agent after a pre-contrast study (Revolution^TM^ Evo, GE Healthcare). Exposition values were as follows: Kv 120, mAs 200, 0.7 s of rotation, 1.250 mm slices, and tilt 0.0. The rabbit was sedated with a protocol involving 0.25 mg/kg Dexmedetomidine (Dextroquillan, 0.5 mg/mL, Fatro S.p.A., Bologna, Italy) and 20 mg/kg ketamine (Nimatek 100 mg/mL, Dechra Veterinary Products S.r.l., Northwich, UK), and then it was placed in an anesthetic induction chamber to avoid injury, stress, and minimizing breath holding. Induction was performed by administering 2 L/h O_2_ and 7% Sevofluorane (Sevofluo 100%, Ecuphar Italia S.r.l., Milano, Italy). Subsequently, the rabbit was positioned in sternal recumbency, and a 24 Gauge venous catheter (Smiths Medical Jelco, Lower Pemberton, Ashford, Kant, UK) was inserted into the right cephalic vein after hair clipping and disinfection for the administration of the contrast agent. The CT examination revealed a nodular oval-shaped mass measuring 7.6 cm × 4.3 cm, occupying a significant portion of the right abdomen with inhomogeneous parenchyma due to the anarchic intralesional vascular branching. The mass exhibited a “whirlpool sign” (Figure 1) on the post-contrast scan, which is an imaging sign indicative of an organ torsion. Its position was consistent with that of the right ovary, which was not visible in the CT study evaluation.

**Figure 2:**
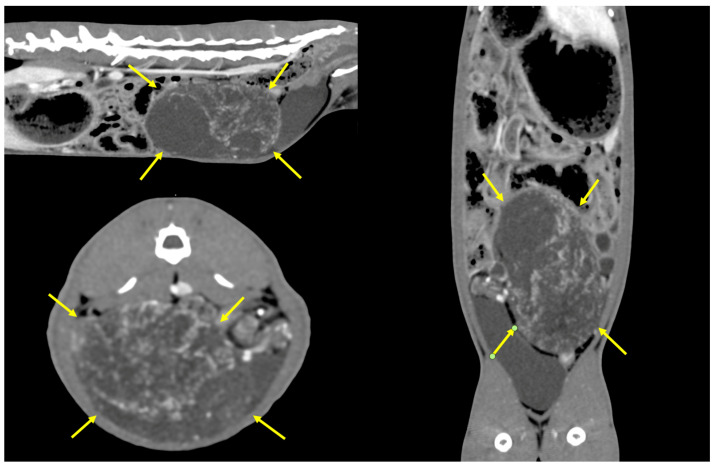
CT scan after contrast agent administration revealed a large dis-homogeneous mass showing low contrast enhancement (yellow arrows), with origin in the right hemiabdomen.

The mass extended from the caudal pole of the left ovary until the dorsal portion of the urinary bladder. The left ovary and uterus were mildly displaced by the mass itself. Possible differential diagnoses included ovarian or uterine neoplasia or a granulomatous peritoneal lesion (Figure 2). No signs of metastatic lesions were detected within the study in the lungs, brain, liver, or bones. Abdominal lymph nodes were unremarkable.

An exploratory laparotomy was conducted following the CT findings. The rabbit was sedated intramuscularly by using Dexmedetomidine (Dextroquillan, 0.5 mg/mL, Fatro S.p.A.), ketamine (Nimatek 100 mg/mL, Dechra Veterinary Products S.r.l.), and methadone (Semfortan 10 mg/mL, Eurovet Animal Health B.V.) at a dosage of 0.25 mg/kg, 20 mg/kg, and 0.2 mg/kg, respectively.

After administration, the rabbit was placed in a heated induction chamber with flow-by oxygen provided. Subsequently, an intravenous catheter was inserted for fluid administration, and intubation was performed on sternal recumbency using a 2.5 mm endotracheal tube assisted by a 30° video-endoscopic optic. Isoflurane (Isovet 1000 mg/mL, Piramal Critical Care Italia S.p.A., San Giovanni Lupatoto, Italy) at a dosage of 0.5% and 1 L/h O_2_ was then administered to maintain anesthesia.

**Figure 3:**
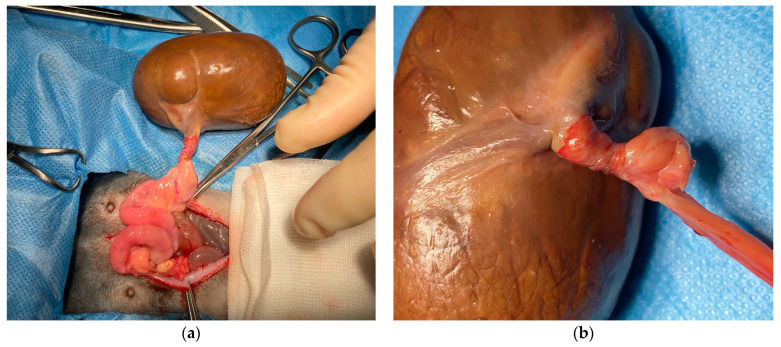
Macroscopic appearance of the mass during the surgical procedure. (**a**) Ovarian mass and its connection to the right uterine horn; (**b**) twisting of the ovarian pedicle and uterine tubes, determining vascular ischemia.

Following anesthesia induction, the rabbit was positioned in dorsal recumbency, and the abdomen was surgically prepared. A 5 cm midline prepubic incision was made using a 21 mm scalpel, and subcutaneous tissues were dissected with forceps until the linea alba was reached and incised. Upon opening the abdomen, a voluminous brown mass with smooth margins and parenchymatous consistency was discovered. Anatomically, the mass corresponded to the right ovary, exhibiting severe torsion of the ovarian pedicle and ligament, along with twisting of the ovarian vessels (Figure 3).

The ovarian artery supplying the mass was meticulously cauterized without complications or bleeding using a technologic Harmonic thermofusion forceps Enseal (Ethicon^TM^ Gen 11, Cincinnati, OH, USA). The same technique was applied to address the contralateral ovary. Subsequently, the broad ligament was dissected to achieve complete visualization of the uterine body and cervix. Prior to the ligation of the vagina, performed with a medium-term absorbable monofilament 3.0 suture, both ureters were located to have a better visualization of the surrounding structures. A thorough exploratory laparotomy was conducted to rule out any coexisting conditions, yielding no remarkable findings. Closure of the fascia and skin was accomplished using a medium-term absorbable 3.0 monofilament thread in a continuous pattern with 1.5 cm spacing. The excised structures were preserved in a plastic container containing 4% formaldehyde for subsequent histopathological examination. Post-surgery, the rabbit received subcutaneous administration of the anti-inflammatory medication Meloxicam (Metacam 5 mg/mL, Boehringer Ingelheim Vetmedica GmbH, Ingelheim am Rhein, Germany), at a dosage of 1 mg/kg. The rabbit experienced a smooth recovery within 24 h following the surgery. Seven days after surgery, the rabbit was presented again to the clinic in good general condition. The suture line did not show swelling, redness, pain, or serum leakage, and abdominal palpation elicited no discomfort. The owner reported an improvement in general behavior and conditions when compared to the pre-surgical period.

Microscopically, diffuse endometrial hyperplasia and vascular congestion of the uterus were found. The right ovary was characterized by diffuse necrosis and few epithelial cells, with no signs of neoplastic transformation detected. The vessels were characterized by the presence of thrombi and necrosis, with an accumulation of fibrin within the interstice. The contralateral ovary appeared normal, with follicles at different stages of maturity and corpora lutea without mitotic activity.

## 3. Discussion

OT refers to the complete or partial rotation of an ovary along the supporting ligaments, resulting in partial or complete ovarian blood flow obstruction, leading to ovarian edema and enlargement. If torsion persists, arterial blood flow is compromised, inducing ischemia and necrosis of adnexal tissue [18]. OT is considered an uncommon but serious condition, accounting for approximately 2.7% of surgical emergencies in women [19,20]. This condition may occur as a consequence of an ovarian mass, such as a teratoma [21,22], other adnexal masses [23], or idiopathically, especially in adolescent age groups [24,25]. In cases where OT occurs in the absence of an ovarian mass, it is believed to be due to congenital elongation of the utero-ovarian ligaments, especially in individuals with a small uterus [18]. While OT is relatively well-documented in human medicine due to its higher incidence, reports and knowledge in veterinary medicine are limited due to the condition’s low prevalence. Nevertheless, isolated cases have been reported in various animal species. In dogs, unilateral OT has been observed secondary to a pyometra [26] and uterine torsion during pregnancy [27]. Two cases of OT have also been described in equines, where the disease occurred as a consequence of a granulosa cell tumor in an adult mare [28] and spontaneously in a female neonatal foal [29]. Other case reports have been described in two unrelated captive iguanas due to bacterial oophoritis and egg retention [30]. Most of the cases described in veterinary medicine report that OT occurs generally secondarily to a previous condition affecting the uterus or ovaries itself. Additionally, OT has been experimentally induced in rabbits in order to evaluate reperfusion injury after surgical correction of the torsion [15,16,17], but spontaneous cases in this group remain undocumented. In the present study, the ovary appeared significantly enlarged, and the twisting of the ovarian artery was clearly evident during surgery. Although no neoplastic tissue was identified upon histological examination due to extensive necrosis, the tumoral etiology of the condition cannot be entirely ruled out. Interestingly, the rabbit did not display any clinical signs of discomfort. However, the torsion occurred along the same axis, involving both the ovarian pedicle and uterine tubes, which likely compromised both inflow and outflow from the affected structures. This could have contributed to the absence of overt clinical symptoms, despite the severity of the torsion. Diagnosis of OT is generally performed in women using ultrasound, CT, or MRI (magnetic resonance imaging), and the whirlpool sign is the most commonly represented characteristic, with a pooled sensitivity of 82% and a specificity of 81% [31]. In cases of OT, the twisted vascular pedicle of the affected ovary results in compromised venous and lymphatic drainage. However, the arterial supply to the ovary remains intact due to the thick and muscular arterial wall, making it incompressible. Differently, the complete torsion, involving both venous and arterial blood flow, observed in the present case may be due to the small vessel’s diameter and the significant weight of the ovary. In women, twisting leads to diffuse ovarian edema, which appears on ultrasound as hypoechoic or heterogeneous stroma with peripheral follicles and subsequent ovarian enlargement. As pressure within the ovary increases over time, arterial blood supply becomes affected, leading to ischemia and infarction [31]. At this stage, Color Doppler examination typically reveals an absent or reduced central blood flow, indicating non-viable tissue. Whirlpool signs are generally found by using ultrasound [31], CT [32], and MRI [33]. In the present study, it was not possible to locate and detect the vascular pedicle with ultrasound, probably due to the high dimensions of the mass. Moreover, a significant reduction in the Color Doppler signal from the affected ovary in rabbits with experimentally induced OT generally occurs after 2 h [34], making it difficult to ascertain the duration of the condition in our case. As reported in women in our study, the whirlpool sign was detected on a CT scan, indicating vascular twisting.

The primitive cause of OT in the present study remains unknown. Ovarian diseases are uncommon in rabbits, and a recent post-mortem study found them to account for 3.3% of all genital tract diseases [14]. A potential explanation for the ovarian enlargement leading to OT could be a neoplastic condition. Ovarian tumors in rabbits are typically benign, including adenomas, luteomas, and hemangiomas, although cases of adenocarcinoma have also been reported [14]. In most reported cases, ovarian neoplasia was an incidental finding in rabbits with uterine adenocarcinoma [12,35,36]. In our study, histopathological examination did not reveal the presence of neoplastic cells within the ovarian mass. However, the necrosis observed in the ovarian parenchyma, resulting from torsion, likely occurred due to ischemia-induced cellular death, masking the underlying cause.

Ovarian necrosis has also been described in rabbits, even though uncommon, with multiple palpable, firm abdominal masses. Histopathologic examination of the reported cases revealed bilateral and extensive necrosis, and only remnants of ovarian tissue were detected in histopathology without evidence of neoplastic transformation or inflammation [37].

## 4. Conclusions

In conclusion, this case report presents the first known instance of spontaneous OT in a rabbit, introducing a novel finding in the field of rabbit medicine. Within the present study, the diagnosis of OT was facilitated by applying imaging techniques such as ultrasound and CT, showing the ability to detect characteristic signs of torsion, including the Whirlpool sign. This study highlights the importance of considering OT as a differential diagnosis in similar clinical presentations, even in young rabbits, and suggests the necessity of broadening the diagnostic focus beyond uterine conditions.

This report emphasizes the need for heightened awareness among veterinary professionals regarding the potential for OT in rabbits, despite its rarity. Future research and case studies are crucial to further investigating the etiology, risk factors, and optimal management strategies for OT in this and other species. Furthermore, this case highlights the critical role of timely diagnosis and intervention in preventing severe complications associated with OT in rabbits and potentially other small mammals.

## Figures and Tables

**Table 1:** Biochemistry profile of the pet rabbit was obtained with a Vetscan2 Abaxis.

Parameter	Result	Reference Range
Albumin	3.5	2.5–5 g/dL
Gamma-glutamyl Transferase	37	18–80 µL
Aspartate Aminotransferase	56	40–70 µL
Amylase	220	200–500 µL
Calcium	14.5	8–14.5 mg/dL
Phosphorus	3.4	2.3–6.9 mg/dL
Total protein	5.88	5.4–7.5 g/dL
Glucose	300	220–350 mg/dL
Sodium	135	133–153 mmol/L
Potassium	4.1	3–45 mmol/L
Globulin	3.6	1.5–3.5 g/dL

## Data Availability

The raw data supporting the conclusions of this article will be made available by the authors upon request.
